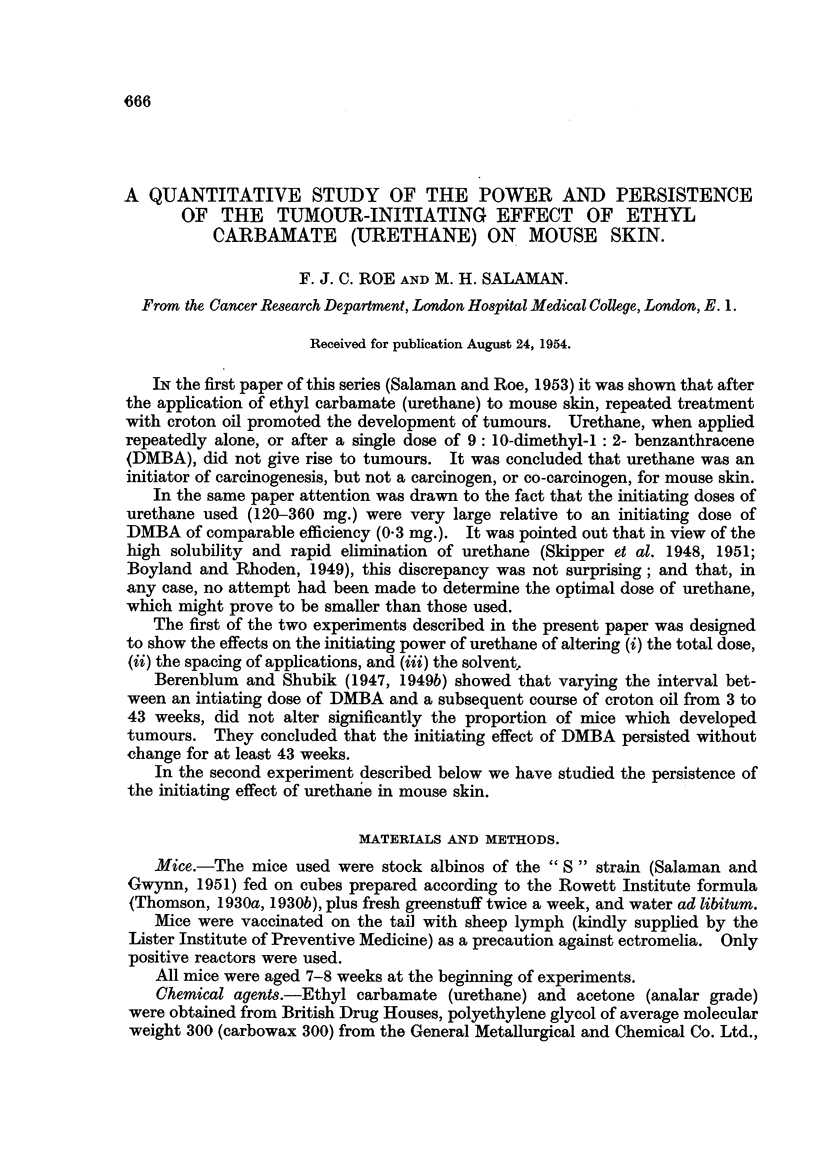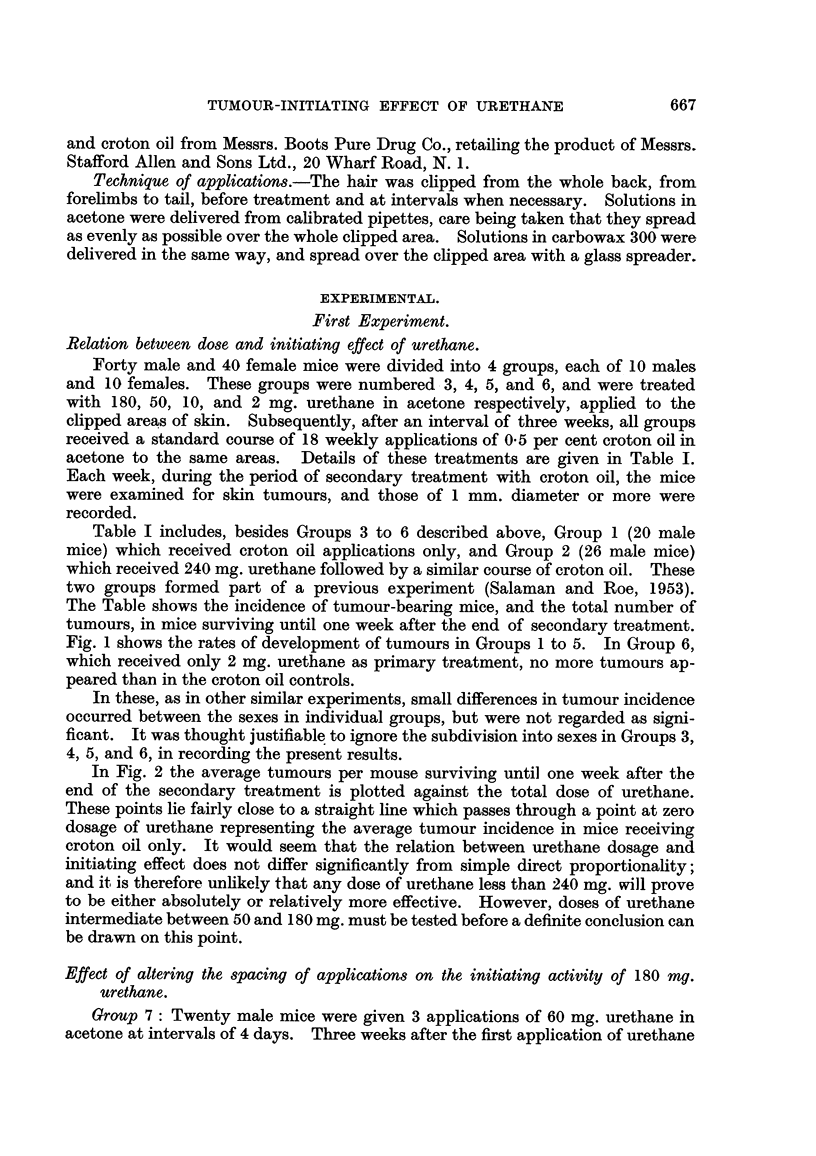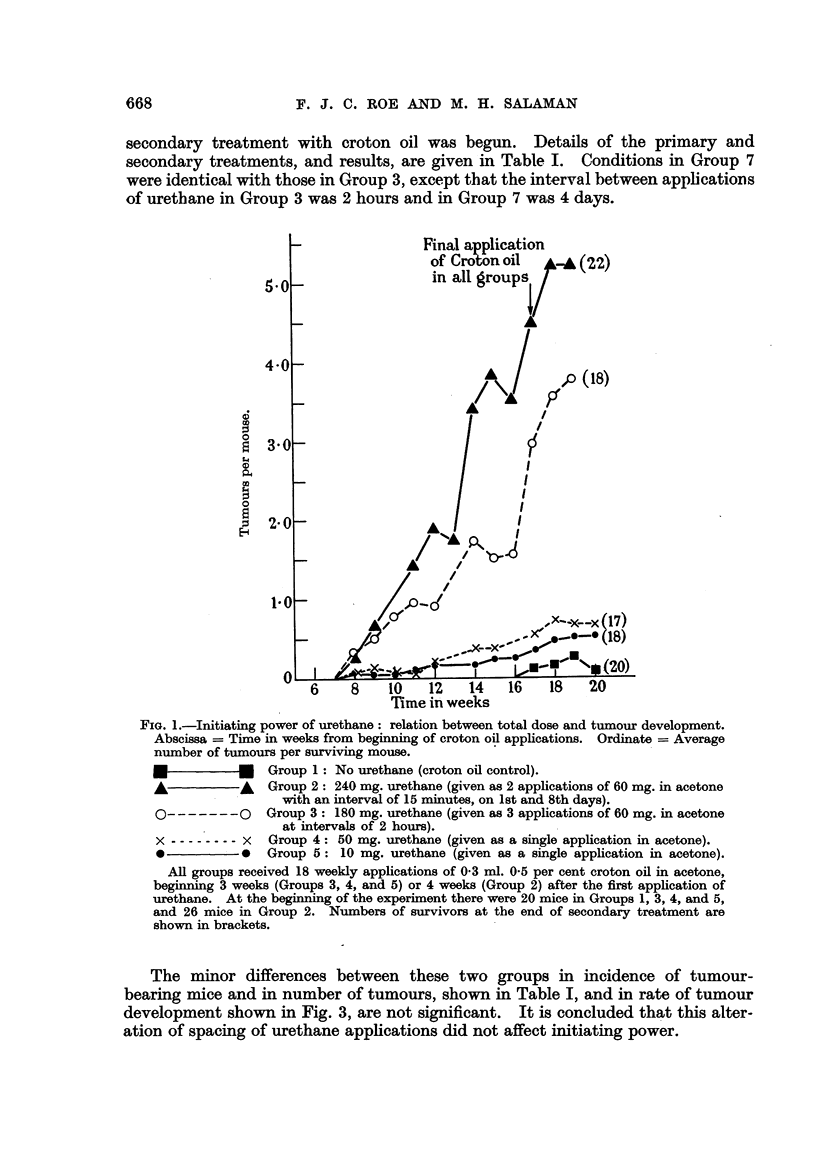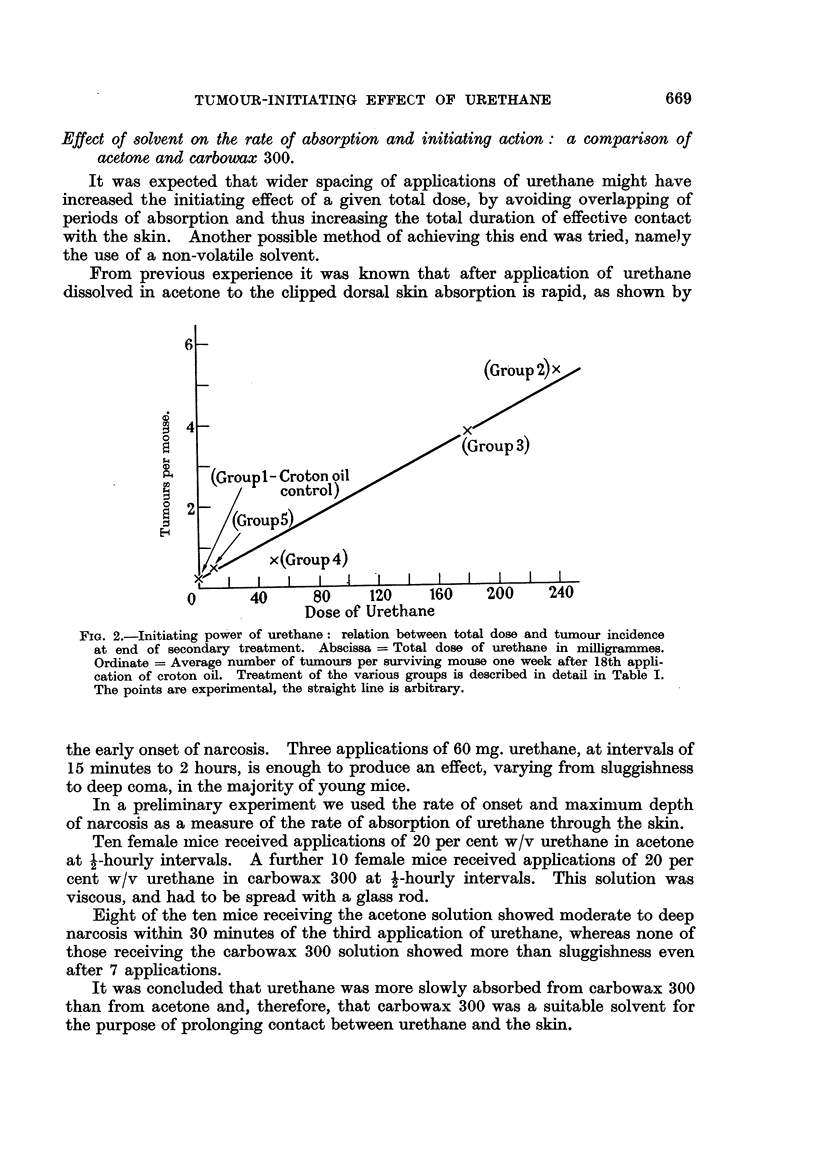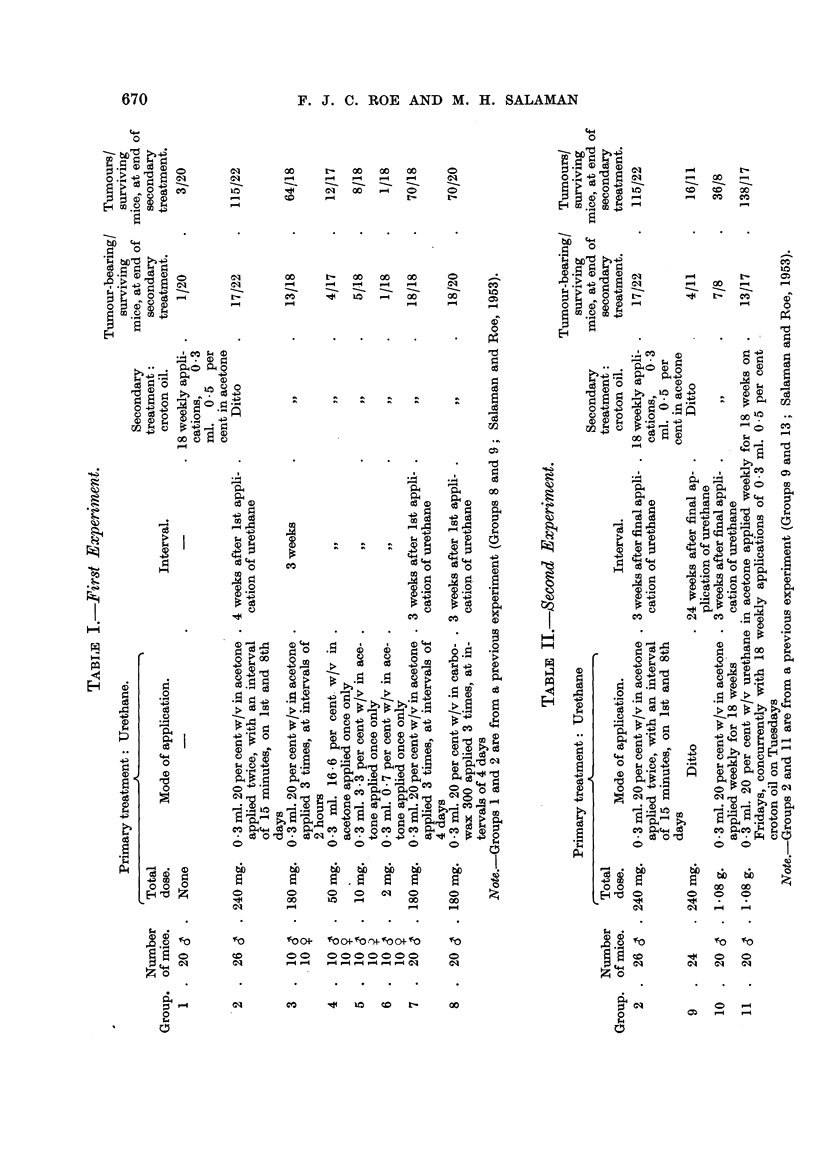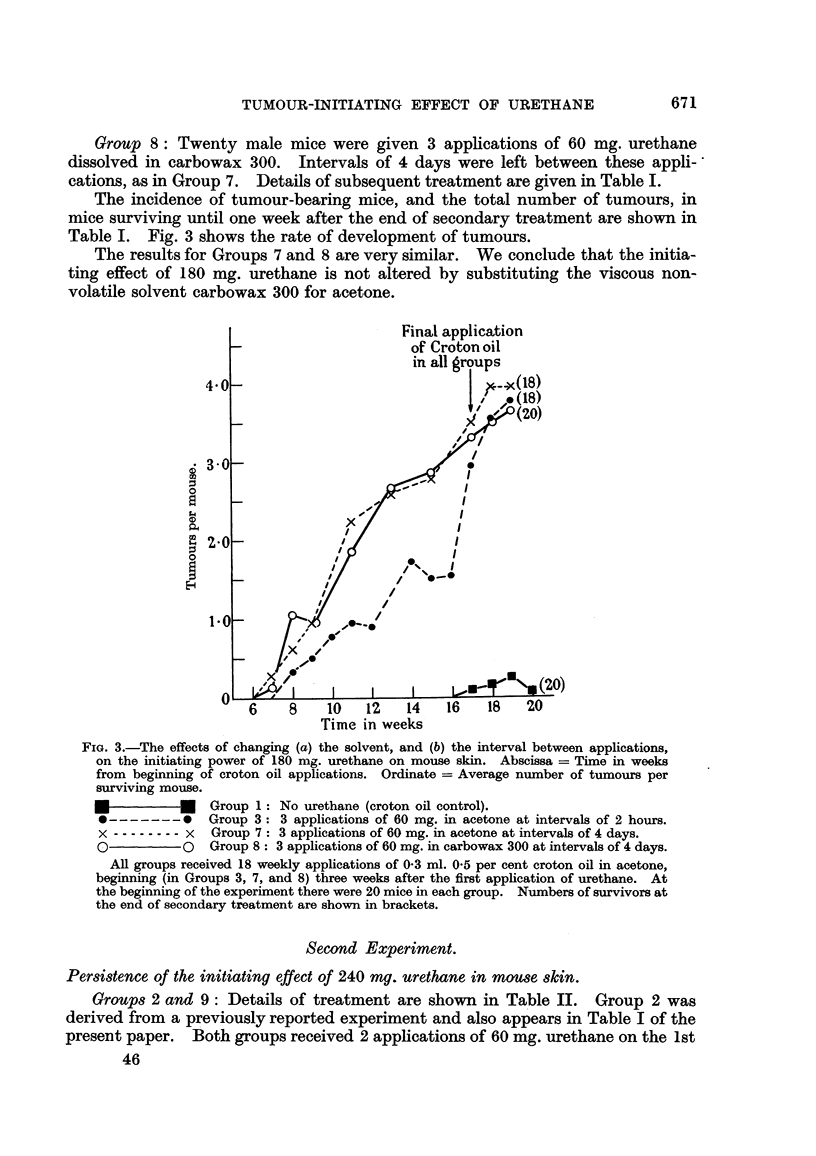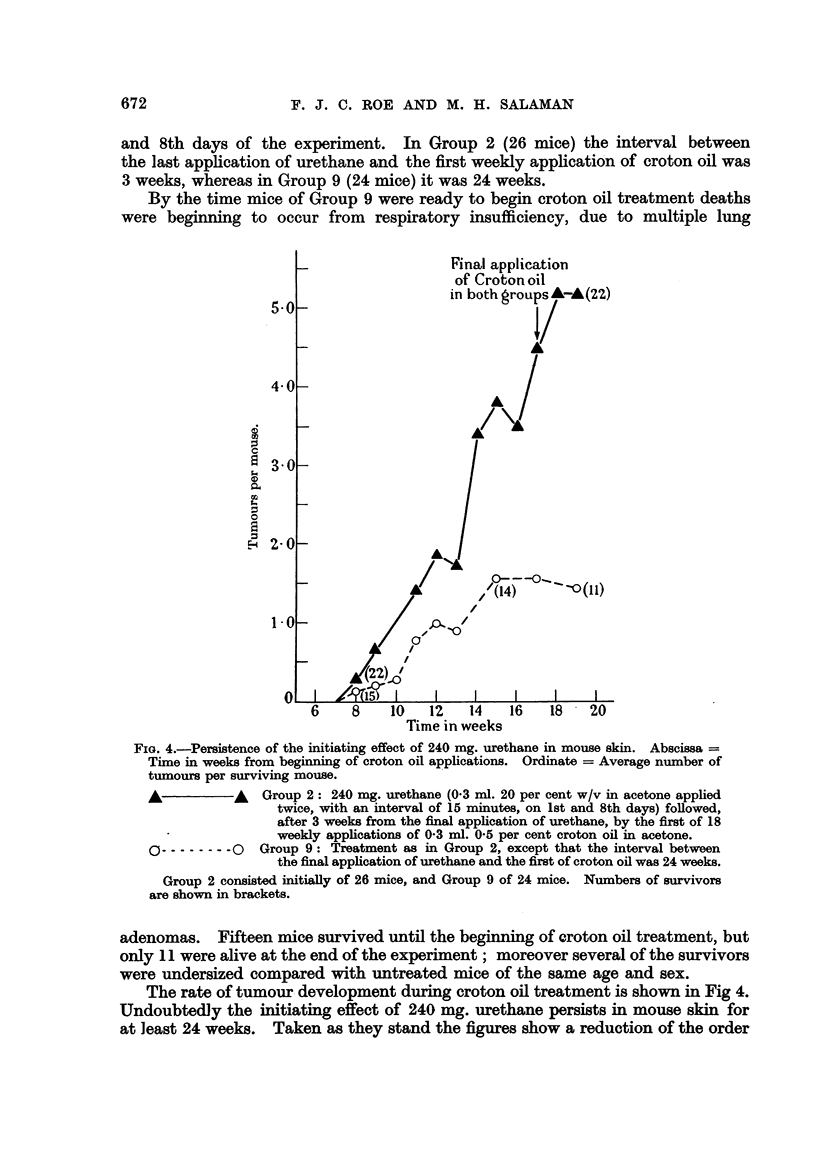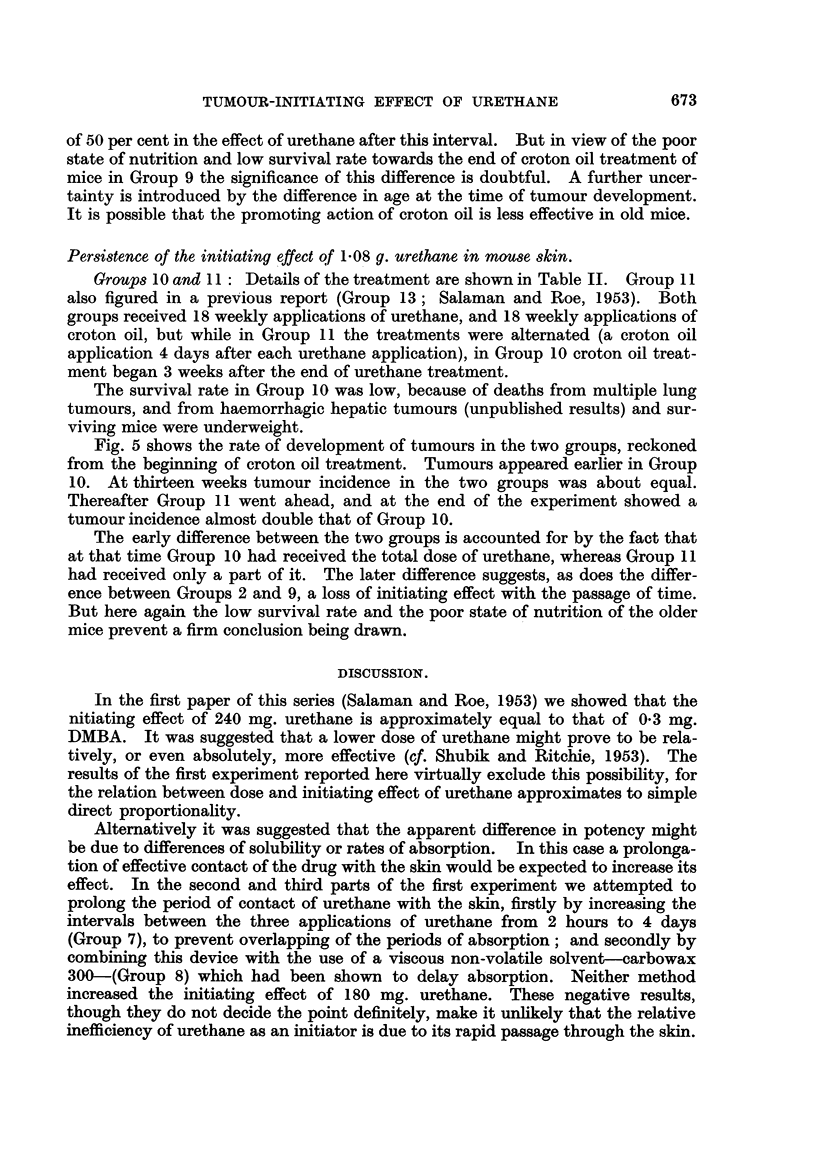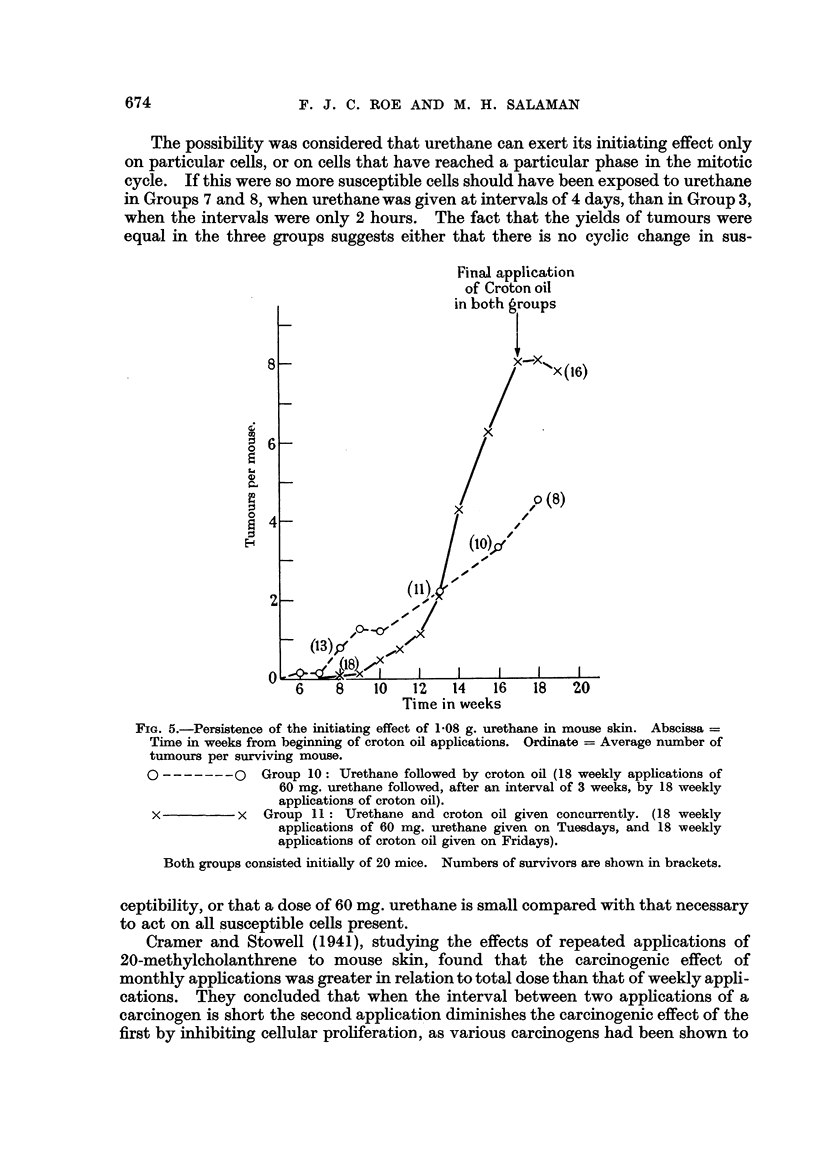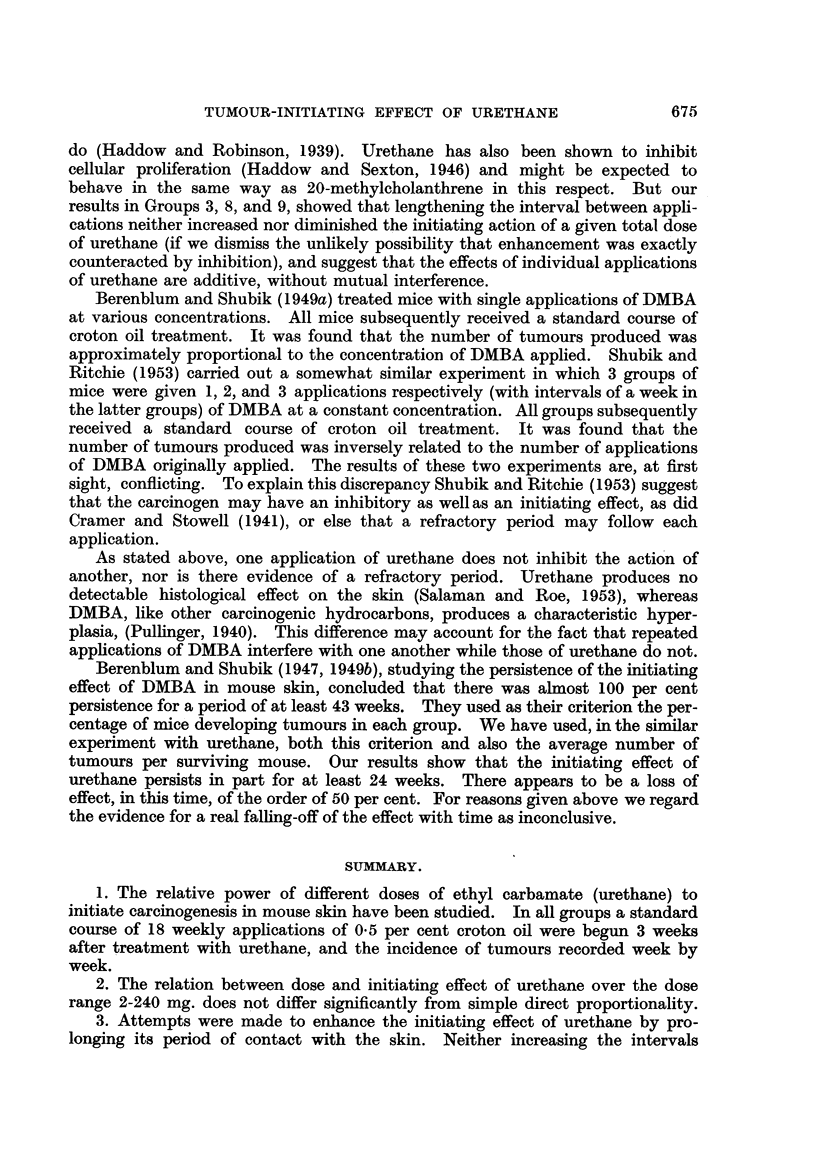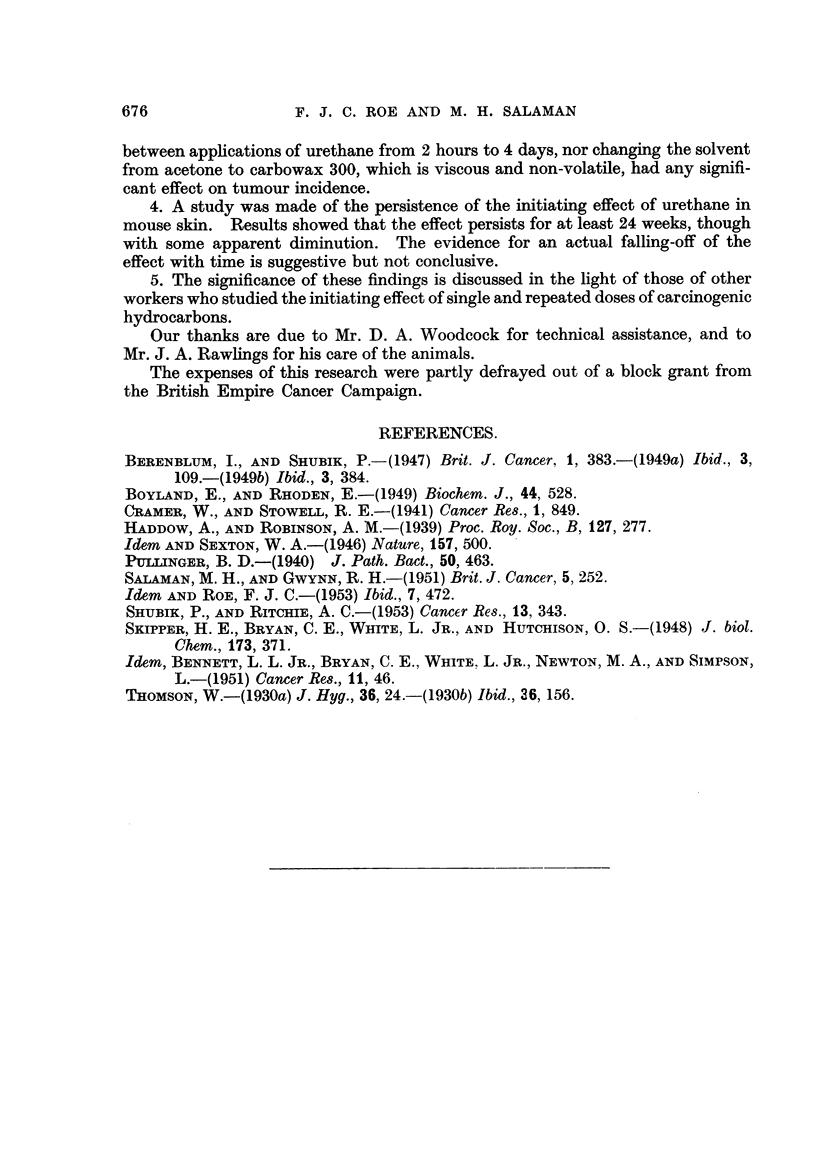# A Quantitative Study of the Power and Persistence of the Tumour-initiating Effect of Ethyl Carbamate (Urethane) on Mouse Skin

**DOI:** 10.1038/bjc.1954.72

**Published:** 1954-12

**Authors:** F. J. C. Roe, M. H. Salaman


### A QUANTITATIVE STUDY OF THE POWER AND PERSISTENCE OF THE TUMOUR-INITIATING EFFECT OF ETHYL CARBAMATE (URETHANE) ON MOUSE SKIN.

F. J. C. ROE AND M. H. SALAMAN.

*From the Cancer Research Department, London Hospital Medical College, London, E. 1.*

Received for publication August 24, 1954.

IN the first paper of this series (Salaman and Roe, 1953) it was shown that after the application of ethyl carbamate (urethane) to mouse skin, repeated treatment with croton oil promoted the development of tumours. Urethane, when applied repeatedly alone, or after a single dose of 9 : 10-dimethyl-1 : 2- benzanthracene (DMBA), did not give rise to tumours. It was concluded that urethane was an initiator of carcinogenesis, but not a carcinogen, or co-carcinogen, for mouse skin.

In the same paper attention was drawn to the fact that the initiating doses of urethane used (120–360 mg.) were very large relative to an initiating dose of DMBA of comparable efficiency (0·3 mg.). It was pointed out that in view of the high solubility and rapid elimination of urethane (Skipper *et al.* 1948, 1951; Boyland and Rhoden, 1949), this discrepancy was not surprising ; and that, in any case, no attempt had been made to determine the optimal dose of urethane, which might prove to be smaller than those used.

The first of the two experiments described in the present paper was designed to show the effects on the initiating power of urethane of altering (*i*) the total dose, (*ii*) the spacing of applications, and (*iii*) the solvent.

Berenblum and Shubik (1947, 1949*b*) showed that varying the interval between an intiating dose of DMBA and a subsequent course of croton oil from 3 to 43 weeks, did not alter significantly the proportion of mice which developed tumours. They concluded that the initiating effect of DMBA persisted without change for at least 43 weeks.

In the second experiment described below we have studied the persistence of the initiating effect of urethane in mouse skin.

### MATERIALS AND METHODS.

*Mice.*—The mice used were stock albinos of the " S " strain (Salaman and Gwynn, 1951) fed on cubes prepared according to the Rowett Institute formula (Thomson, 1930*a*, 1930*b*), plus fresh greenstuff twice a week, and water *ad libitum.*

Mice were vaccinated on the tail with sheep lymph (kindly supplied by the Lister Institute of Preventive Medicine) as a precaution against ectromelia. Only positive reactors were used.

All mice were aged 7–8 weeks at the beginning of experiments.

*Chemical agents.*—Ethyl carbamate (urethane) and acetone (analar grade) were obtained from British Drug Houses, polyethylene glycol of average molecular weight 300 (carbowax 300) from the General Metallurgical and Chemical Co. Ltd., and croton oil from Messrs. Boots Pure Drug Co., retailing the product of Messrs. Stafford Allen and Sons Ltd., 20 Wharf Road, N. 1.

*Technique of applications.*—The hair was clipped from the whole back, from forelimbs to tail, before treatment and at intervals when necessary. Solutions in acetone were delivered from calibrated pipettes, care being taken that they spread as evenly as possible over the whole clipped area. Solutions in carbowax 300 were delivered in the same way, and spread over the clipped area with a glass spreader.

### EXPERIMENTAL.

### *First Experiment.*

*Relation between dose and initiating effect of urethane.*

Forty male and 40 female mice were divided into 4 groups, each of 10 males and 10 females. These groups were numbered 3, 4, 5, and 6, and were treated with 180, 50, 10, and 2 mg. urethane in acetone respectively, applied to the clipped areas of skin. Subsequently, after an interval of three weeks, all groups received a standard course of 18 weekly applications of 0·5 per cent croton oil in acetone to the same areas. Details of these treatments are given in Table I. Each week, during the period of secondary treatment with croton oil, the mice were examined for skin tumours, and those of 1 mm. diameter or more were recorded.

Table I includes, besides Groups 3 to 6 described above, Group 1 (20 male mice) which received croton oil applications only, and Group 2 (26 male mice) which received 240 mg. urethane followed by a similar course of croton oil. These two groups formed part of a previous experiment (Salaman and Roe, 1953). The Table shows the incidence of tumour-bearing mice, and the total number of tumours, in mice surviving until one week after the end of secondary treatment. Fig. 1 shows the rates of development of tumours in Groups 1 to 5. In Group 6, which received only 2 mg. urethane as primary treatment, no more tumours appeared than in the croton oil controls.

In these, as in other similar experiments, small differences in tumour incidence occurred between the sexes in individual groups, but were not regarded as significant. It was thought justifiable to ignore the subdivision into sexes in Groups 3, 4, 5, and 6, in recording the present results.

In Fig. 2 the average tumours per mouse surviving until one week after the end of the secondary treatment is plotted against the total dose of urethane. These points lie fairly close to a straight line which passes through a point at zero dosage of urethane representing the average tumour incidence in mice receiving croton oil only. It would seem that the relation between urethane dosage and initiating effect does not differ significantly from simple direct proportionality; and it is therefore unlikely that any dose of urethane less than 240 mg. will prove to be either absolutely or relatively more effective. However, doses of urethane intermediate between 50 and 180 mg. must be tested before a definite conclusion can be drawn on this point.

*Effect of altering the spacing of applications on the initiating activity of* 180 *mg. urethane.*

*Group* 7 : Twenty male mice were given 3 applications of 60 mg. urethane in acetone at intervals of 4 days. Three weeks after the first application of urethane secondary treatment with croton oil was begun. Details of the primary and secondary treatments, and results, are given in Table I. Conditions in Group 7 were identical with those in Group 3, except that the interval between applications of urethane in Group 3 was 2 hours and in Group 7 was 4 days.

FIG. 1.—Initiating power of urethane : relation between total dose and tumour development. Abscissa = Time in weeks from beginning of croton oil applications. Ordinate = Average number of tumours per surviving mouse.

■————■ Group 1 : No urethane (croton oil control).
▲————▲ Group 2 : 240 mg. urethane (given as 2 applications of 60 mg. in acetone with an interval of 15 minutes, on 1st and 8th days).
○- - - - - - -○ Group 3 : 180 mg. urethane (given as 3 applications of 60 mg. in acetone at intervals of 2 hours).
× - - - - - - - × Group 4 : 50 mg. urethane (given as a single application in acetone).
●————● Group 5 : 10 mg. urethane (given as a single application in acetone).

All groups received 18 weekly applications of 0·3 ml. 0·5 per cent croton oil in acetone, beginning 3 weeks (Groups 3, 4, and 5) or 4 weeks (Group 2) after the first application of urethane. At the beginning of the experiment there were 20 mice in Groups 1, 3, 4, and 5, and 26 mice in Group 2. Numbers of survivors at the end of secondary treatment are shown in brackets.

The minor differences between these two groups in incidence of tumour-bearing mice and in number of tumours, shown in Table I, and in rate of tumour development shown in Fig. 3, are not significant. It is concluded that this alteration of spacing of urethane applications did not affect initiating power.

*Effect of solvent on the rate of absorption and initiating action: a comparison of acetone and carbowax* 300.

It was expected that wider spacing of applications of urethane might have increased the initiating effect of a given total dose, by avoiding overlapping of periods of absorption and thus increasing the total duration of effective contact with the skin. Another possible method of achieving this end was tried, namely the use of a non-volatile solvent.

From previous experience it was known that after application of urethane dissolved in acetone to the clipped dorsal skin absorption is rapid, as shown by

Fig. 2.—Initiating power of urethane: relation between total dose and tumour incidence at end of secondary treatment. Abscissa = Total dose of urethane in milligrammes. Ordinate = Average number of tumours per surviving mouse one week after 18th application of croton oil. Treatment of the various groups is described in detail in Table I. The points are experimental, the straight line is arbitrary.

the early onset of narcosis. Three applications of 60 mg. urethane, at intervals of 15 minutes to 2 hours, is enough to produce an effect, varying from sluggishness to deep coma, in the majority of young mice.

In a preliminary experiment we used the rate of onset and maximum depth of narcosis as a measure of the rate of absorption of urethane through the skin.

Ten female mice received applications of 20 per cent w/v urethane in acetone at ½-hourly intervals. A further 10 female mice received applications of 20 per cent w/v urethane in carbowax 300 at ½-hourly intervals. This solution was viscous, and had to be spread with a glass rod.

Eight of the ten mice receiving the acetone solution showed moderate to deep narcosis within 30 minutes of the third application of urethane, whereas none of those receiving the carbowax 300 solution showed more than sluggishness even after 7 applications.

It was concluded that urethane was more slowly absorbed from carbowax 300 than from acetone and, therefore, that carbowax 300 was a suitable solvent for the purpose of prolonging contact between urethane and the skin.

TABLE I.—*First Experiment.*

| Group. | Number of mice. | Primary treatment: Urethane. Total dose. | Mode of application. | Interval. | Secondary treatment: croton oil. | Tumour-bearing/surviving mice, at end of secondary treatment. | Tumours/surviving mice, at end of secondary treatment. |
|---|---|---|---|---|---|---|---|
| 1 | 20 ♂ | None | — | — | 18 weekly applications, 0·3 ml. 0·5 per cent in acetone | 1/20 | 3/20 |
| 2 | 26 ♂ | 240 mg. | 0·3 ml. 20 per cent w/v in acetone applied twice, with an interval of 15 minutes, on 1st and 8th days | 4 weeks after 1st application of urethane | Ditto | 17/22 | 115/22 |
| 3 | 10 ♂<br>10 ♀ | 180 mg. | 0·3 ml. 20 per cent w/v in acetone applied 3 times, at intervals of 2 hours | 3 weeks | „ | 13/18 | 64/18 |
| 4 | 10 ♂<br>10 ♀ | 50 mg. | 0·3 ml. 16·6 per cent w/v in acetone applied once only | „ | „ | 4/17 | 12/17 |
| 5 | 10 ♂<br>10 ♀ | 10 mg. | 0·3 ml. 3·3 per cent w/v in acetone applied once only | „ | „ | 5/18 | 8/18 |
| 6 | 10 ♂<br>10 ♀ | 2 mg. | 0·3 ml. 0·7 per cent w/v in acetone applied once only | „ | „ | 1/18 | 1/18 |
| 7 | 20 ♂ | 180 mg. | 0·3 ml. 20 per cent w/v in acetone applied 3 times, at intervals of 4 days | 3 weeks after 1st application of urethane | „ | 18/18 | 70/18 |
| 8 | 20 ♂ | 180 mg. | 0·3 ml. 20 per cent w/v in carbowax 300 applied 3 times, at intervals of 4 days | 3 weeks after 1st application of urethane | „ | 18/20 | 70/20 |

*Note.*—Groups 1 and 2 are from a previous experiment (Groups 8 and 9; Salaman and Roe, 1953).

TABLE II.—*Second Experiment.*

| Group. | Number of mice. | Primary treatment: Urethane Total dose. | Mode of application. | Interval. | Secondary treatment: croton oil. | Tumour-bearing/surviving mice, at end of secondary treatment. | Tumours/surviving mice, at end of secondary treatment. |
|---|---|---|---|---|---|---|---|
| 2 | 26 ♂ | 240 mg. | 0·3 ml. 20 per cent w/v in acetone applied twice, with an interval of 15 minutes, on 1st and 8th days | 3 weeks after final application of urethane | 18 weekly applications, 0·3 ml. 0·5 per cent in acetone | 17/22 | 115/22 |
| 9 | 24 | 240 mg. | Ditto | 24 weeks after final application of urethane | Ditto | 4/11 | 16/11 |
| 10 | 20 ♂ | 1·08 g. | 0·3 ml. 20 per cent w/v in acetone applied weekly for 18 weeks | 3 weeks after final application of urethane | „ | 7/8 | 36/8 |
| 11 | 20 ♂ | 1·08 g. | 0·3 ml. 20 per cent w/v urethane in acetone applied weekly for 18 weeks on Fridays, concurrently with 18 weekly applications of 0·3 ml. 0·5 per cent croton oil on Tuesdays | | | 13/17 | 138/17 |

*Note.*—Groups 2 and 11 are from a previous experiment (Groups 9 and 13; Salaman and Roe, 1953).

*Group* 8 : Twenty male mice were given 3 applications of 60 mg. urethane dissolved in carbowax 300. Intervals of 4 days were left between these applications, as in Group 7. Details of subsequent treatment are given in Table I.

The incidence of tumour-bearing mice, and the total number of tumours, in mice surviving until one week after the end of secondary treatment are shown in Table I. Fig. 3 shows the rate of development of tumours.

The results for Groups 7 and 8 are very similar. We conclude that the initiating effect of 180 mg. urethane is not altered by substituting the viscous non-volatile solvent carbowax 300 for acetone.

FIG. 3.—The effects of changing (*a*) the solvent, and (*b*) the interval between applications, on the initiating power of 180 mg. urethane on mouse skin. Abscissa = Time in weeks from beginning of croton oil applications. Ordinate = Average number of tumours per surviving mouse.

■———■ Group 1 : No urethane (croton oil control).
●- - - - - -● Group 3 : 3 applications of 60 mg. in acetone at intervals of 2 hours.
× - - - - - - × Group 7 : 3 applications of 60 mg. in acetone at intervals of 4 days.
○———○ Group 8 : 3 applications of 60 mg. in carbowax 300 at intervals of 4 days.

All groups received 18 weekly applications of 0·3 ml. 0·5 per cent croton oil in acetone, beginning (in Groups 3, 7, and 8) three weeks after the first application of urethane. At the beginning of the experiment there were 20 mice in each group. Numbers of survivors at the end of secondary treatment are shown in brackets.

### *Second Experiment.*

*Persistence of the initiating effect of* 240 *mg. urethane in mouse skin.*

*Groups* 2 *and* 9 : Details of treatment are shown in Table II. Group 2 was derived from a previously reported experiment and also appears in Table I of the present paper. Both groups received 2 applications of 60 mg. urethane on the 1st and 8th days of the experiment. In Group 2 (26 mice) the interval between the last application of urethane and the first weekly application of croton oil was 3 weeks, whereas in Group 9 (24 mice) it was 24 weeks.

By the time mice of Group 9 were ready to begin croton oil treatment deaths were beginning to occur from respiratory insufficiency, due to multiple lung

Fig. 4.—Persistence of the initiating effect of 240 mg. urethane in mouse skin. Abscissa = Time in weeks from beginning of croton oil applications. Ordinate = Average number of tumours per surviving mouse.

▲———▲ Group 2 : 240 mg. urethane (0·3 ml. 20 per cent w/v in acetone applied twice, with an interval of 15 minutes, on 1st and 8th days) followed, after 3 weeks from the final application of urethane, by the first of 18 weekly applications of 0·3 ml. 0·5 per cent croton oil in acetone.

○- - - - - - -○ Group 9 : Treatment as in Group 2, except that the interval between the final application of urethane and the first of croton oil was 24 weeks.

Group 2 consisted initially of 26 mice, and Group 9 of 24 mice. Numbers of survivors are shown in brackets.

adenomas. Fifteen mice survived until the beginning of croton oil treatment, but only 11 were alive at the end of the experiment ; moreover several of the survivors were undersized compared with untreated mice of the same age and sex.

The rate of tumour development during croton oil treatment is shown in Fig 4. Undoubtedly the initiating effect of 240 mg. urethane persists in mouse skin for at least 24 weeks. Taken as they stand the figures show a reduction of the order of 50 per cent in the effect of urethane after this interval. But in view of the poor state of nutrition and low survival rate towards the end of croton oil treatment of mice in Group 9 the significance of this difference is doubtful. A further uncertainty is introduced by the difference in age at the time of tumour development. It is possible that the promoting action of croton oil is less effective in old mice.

*Persistence of the initiating effect of* 1·08 *g. urethane in mouse skin.*

*Groups* 10 *and* 11 : Details of the treatment are shown in Table II. Group 11 also figured in a previous report (Group 13 ; Salaman and Roe, 1953). Both groups received 18 weekly applications of urethane, and 18 weekly applications of croton oil, but while in Group 11 the treatments were alternated (a croton oil application 4 days after each urethane application), in Group 10 croton oil treatment began 3 weeks after the end of urethane treatment.

The survival rate in Group 10 was low, because of deaths from multiple lung tumours, and from haemorrhagic hepatic tumours (unpublished results) and surviving mice were underweight.

Fig. 5 shows the rate of development of tumours in the two groups, reckoned from the beginning of croton oil treatment. Tumours appeared earlier in Group 10. At thirteen weeks tumour incidence in the two groups was about equal. Thereafter Group 11 went ahead, and at the end of the experiment showed a tumour incidence almost double that of Group 10.

The early difference between the two groups is accounted for by the fact that at that time Group 10 had received the total dose of urethane, whereas Group 11 had received only a part of it. The later difference suggests, as does the difference between Groups 2 and 9, a loss of initiating effect with the passage of time. But here again the low survival rate and the poor state of nutrition of the older mice prevent a firm conclusion being drawn.

### DISCUSSION.

In the first paper of this series (Salaman and Roe, 1953) we showed that the nitiating effect of 240 mg. urethane is approximately equal to that of 0·3 mg. DMBA. It was suggested that a lower dose of urethane might prove to be relatively, or even absolutely, more effective (*cf.* Shubik and Ritchie, 1953). The results of the first experiment reported here virtually exclude this possibility, for the relation between dose and initiating effect of urethane approximates to simple direct proportionality.

Alternatively it was suggested that the apparent difference in potency might be due to differences of solubility or rates of absorption. In this case a prolongation of effective contact of the drug with the skin would be expected to increase its effect. In the second and third parts of the first experiment we attempted to prolong the period of contact of urethane with the skin, firstly by increasing the intervals between the three applications of urethane from 2 hours to 4 days (Group 7), to prevent overlapping of the periods of absorption ; and secondly by combining this device with the use of a viscous non-volatile solvent—carbowax 300—(Group 8) which had been shown to delay absorption. Neither method increased the initiating effect of 180 mg. urethane. These negative results, though they do not decide the point definitely, make it unlikely that the relative inefficiency of urethane as an initiator is due to its rapid passage through the skin.

The possibility was considered that urethane can exert its initiating effect only on particular cells, or on cells that have reached a particular phase in the mitotic cycle. If this were so more susceptible cells should have been exposed to urethane in Groups 7 and 8, when urethane was given at intervals of 4 days, than in Group 3, when the intervals were only 2 hours. The fact that the yields of tumours were equal in the three groups suggests either that there is no cyclic change in sus-

FIG. 5.—Persistence of the initiating effect of 1·08 g. urethane in mouse skin. Abscissa = Time in weeks from beginning of croton oil applications. Ordinate = Average number of tumours per surviving mouse.

O - - - - - - - O Group 10 : Urethane followed by croton oil (18 weekly applications of 60 mg. urethane followed, after an interval of 3 weeks, by 18 weekly applications of croton oil).

× ——————— × Group 11 : Urethane and croton oil given concurrently. (18 weekly applications of 60 mg. urethane given on Tuesdays, and 18 weekly applications of croton oil given on Fridays).

Both groups consisted initially of 20 mice. Numbers of survivors are shown in brackets.

ceptibility, or that a dose of 60 mg. urethane is small compared with that necessary to act on all susceptible cells present.

Cramer and Stowell (1941), studying the effects of repeated applications of 20-methylcholanthrene to mouse skin, found that the carcinogenic effect of monthly applications was greater in relation to total dose than that of weekly applications. They concluded that when the interval between two applications of a carcinogen is short the second application diminishes the carcinogenic effect of the first by inhibiting cellular proliferation, as various carcinogens had been shown to do (Haddow and Robinson, 1939). Urethane has also been shown to inhibit cellular proliferation (Haddow and Sexton, 1946) and might be expected to behave in the same way as 20-methylcholanthrene in this respect. But our results in Groups 3, 8, and 9, showed that lengthening the interval between applications neither increased nor diminished the initiating action of a given total dose of urethane (if we dismiss the unlikely possibility that enhancement was exactly counteracted by inhibition), and suggest that the effects of individual applications of urethane are additive, without mutual interference.

Berenblum and Shubik (1949*a*) treated mice with single applications of DMBA at various concentrations. All mice subsequently received a standard course of croton oil treatment. It was found that the number of tumours produced was approximately proportional to the concentration of DMBA applied. Shubik and Ritchie (1953) carried out a somewhat similar experiment in which 3 groups of mice were given 1, 2, and 3 applications respectively (with intervals of a week in the latter groups) of DMBA at a constant concentration. All groups subsequently received a standard course of croton oil treatment. It was found that the number of tumours produced was inversely related to the number of applications of DMBA originally applied. The results of these two experiments are, at first sight, conflicting. To explain this discrepancy Shubik and Ritchie (1953) suggest that the carcinogen may have an inhibitory as well as an initiating effect, as did Cramer and Stowell (1941), or else that a refractory period may follow each application.

As stated above, one application of urethane does not inhibit the action of another, nor is there evidence of a refractory period. Urethane produces no detectable histological effect on the skin (Salaman and Roe, 1953), whereas DMBA, like other carcinogenic hydrocarbons, produces a characteristic hyperplasia, (Pullinger, 1940). This difference may account for the fact that repeated applications of DMBA interfere with one another while those of urethane do not.

Berenblum and Shubik (1947, 1949*b*), studying the persistence of the initiating effect of DMBA in mouse skin, concluded that there was almost 100 per cent persistence for a period of at least 43 weeks. They used as their criterion the percentage of mice developing tumours in each group. We have used, in the similar experiment with urethane, both this criterion and also the average number of tumours per surviving mouse. Our results show that the initiating effect of urethane persists in part for at least 24 weeks. There appears to be a loss of effect, in this time, of the order of 50 per cent. For reasons given above we regard the evidence for a real falling-off of the effect with time as inconclusive.

### SUMMARY.

1. The relative power of different doses of ethyl carbamate (urethane) to initiate carcinogenesis in mouse skin have been studied. In all groups a standard course of 18 weekly applications of 0·5 per cent croton oil were begun 3 weeks after treatment with urethane, and the incidence of tumours recorded week by week.

2. The relation between dose and initiating effect of urethane over the dose range 2-240 mg. does not differ significantly from simple direct proportionality.

3. Attempts were made to enhance the initiating effect of urethane by prolonging its period of contact with the skin. Neither increasing the intervals between applications of urethane from 2 hours to 4 days, nor changing the solvent from acetone to carbowax 300, which is viscous and non-volatile, had any significant effect on tumour incidence.

4. A study was made of the persistence of the initiating effect of urethane in mouse skin. Results showed that the effect persists for at least 24 weeks, though with some apparent diminution. The evidence for an actual falling-off of the effect with time is suggestive but not conclusive.

5. The significance of these findings is discussed in the light of those of other workers who studied the initiating effect of single and repeated doses of carcinogenic hydrocarbons.

Our thanks are due to Mr. D. A. Woodcock for technical assistance, and to Mr. J. A. Rawlings for his care of the animals.

The expenses of this research were partly defrayed out of a block grant from the British Empire Cancer Campaign.

### REFERENCES.

BERENBLUM, I., AND SHUBIK, P.—(1947) *Brit. J. Cancer*, **1**, 383.—(1949*a*) *Ibid.*, **3**, 109.—(1949*b*) *Ibid.*, **3**, 384.
BOYLAND, E., AND RHODEN, E.—(1949) *Biochem. J.*, **44**, 528.
CRAMER, W., AND STOWELL, R. E.—(1941) *Cancer Res.*, **1**, 849.
HADDOW, A., AND ROBINSON, A. M.—(1939) *Proc. Roy. Soc.*, *B*, **127**, 277.
*Idem* AND SEXTON, W. A.—(1946) *Nature*, **157**, 500.
PULLINGER, B. D.—(1940) *J. Path. Bact.*, **50**, 463.
SALAMAN, M. H., AND GWYNN, R. H.—(1951) *Brit. J. Cancer*, **5**, 252.
*Idem* AND ROE, F. J. C.—(1953) *Ibid.*, **7**, 472.
SHUBIK, P., AND RITCHIE, A. C.—(1953) *Cancer Res.*, **13**, 343.
SKIPPER, H. E., BRYAN, C. E., WHITE, L. JR., AND HUTCHISON, O. S.—(1948) *J. biol. Chem.*, **173**, 371.
*Idem*, BENNETT, L. L. JR., BRYAN, C. E., WHITE, L. JR., NEWTON, M. A., AND SIMPSON, L.—(1951) *Cancer Res.*, **11**, 46.
THOMSON, W.—(1930*a*) *J. Hyg.*, **36**, 24.—(1930*b*) *Ibid.*, **36**, 156.